# Comparative analysis of flavivirus sfRNA dynamics and secondary structure

**DOI:** 10.1128/jvi.00898-26

**Published:** 2026-07-10

**Authors:** Camden R. Bair, David VanInsberghe, Anice C. Lowen, Graeme L. Conn, Christopher J. Neufeldt

**Affiliations:** 1 Department of Microbiology and Immunology, Emory University School of Medicine 12239, Atlanta, Georgia, USA; 2 Department of Biochemistry, Emory University School of Medicine 12239, Atlanta, Georgia, USA; The University of Texas Southwestern Medical Center, Dallas, Texas, USA

**Keywords:** flavivirus, *Orthoflavivirus*, sfRNA, SHAPE-MaP, RNA secondary structure

## Abstract

The accumulation of subgenomic flavivirus RNAs (sfRNAs) modulates viral fitness and pathogenicity in culture and *in vivo*. These noncoding RNAs are produced by incomplete digestion of the flavivirus genome by the cellular 5′−3′ exoribonuclease (XRN1). Diverse flaviviruses have conserved RNA structural elements (RSEs) that map to their 3′-untranslated region (3′-UTR): Xrn-resistant RNA structures, dumbbell structures, and a 3′-stem loop (3′-SL). Despite the importance of the 3′-UTR RSEs for flavivirus replication, the structural dynamics of sfRNA during flavivirus infection are understudied. Here, we use digital droplet PCR to quantify sfRNA levels during infection for a panel of mosquito-borne flaviviruses, including dengue virus serotypes 1 (DENV1), 2 (DENV2), and 4 (DENV4), and Zika virus. We then used SHAPE-MaP on XRN1-digested, *in vitro*-transcribed sfRNAs from each virus to determine their secondary structures compared to the corresponding sfRNAs obtained from flavivirus-infected A549 cells. Results seen in-cell and *in vitro* were largely similar; however, motifs within the dumbbell, the small hairpin directly upstream of the 3′-SL, and 3′-SL regions showed significant differences in the extent of nucleotide reactivity. These differences were consistent among the four flaviviruses examined and may indicate regions of sfRNA that are shielded by interaction with proteins or other nucleic acids during infection. However, strong protection indicative of sustained interaction was not apparent. Our findings suggest that sfRNA interactions with viral and host factors within the cell are few, occur via base-paired regions, or are highly transient.

**IMPORTANCE:**

Flaviviruses are highly prevalent human pathogens. The flavivirus genome contains RNA structural elements (RSEs), including those encoded in the 3′-UTR, that are necessary for viral replication. Subgenomic flavivirus RNAs (sfRNAs) are produced by incomplete digestion of flavivirus genomic RNA due to the cellular exoribonuclease XRN1 encountering 3′-UTR RSEs that promote XRN1 stalling and disassociation. Viruses unable to produce sfRNAs are highly attenuated, underlining their biological importance. sfRNA secondary structure has been investigated previously, but little information is available on sfRNA secondary structure dynamics in infected cells. By comparing SHAPE-MaP reactivities *in vitro* and in cells, we determined that previously inferred structures are likely maintained within infected cells. We also identified differences in the extent of SHAPE reactivity between *in vitro* and in-cell environments that were common to multiple mosquito-borne flaviviruses. These differences suggest that sfRNAs may engage in transient interactions within the cell that may be important for their function.

## INTRODUCTION

Flaviviruses (genus *Orthoflavivirus*) are arthropod-borne viruses that pose a major global threat to human health (1). Infections by dengue virus alone cause an estimated 100–400 million infections yearly, with endemic circulation in more than 100 countries across Latin America, Africa, Asia, and Oceania (2, 3). Other flaviviruses such as West Nile virus, Zika virus (ZIKV), Japanese encephalitis virus, and yellow fever virus also impact global health (4, 5). Owing to a lack of antivirals, limited availability or efficacy of vaccines, and shifts in vector ecology driven by environmental change, flaviviruses continue to pose a serious threat to human health (6, 7).

**Fig 1:**
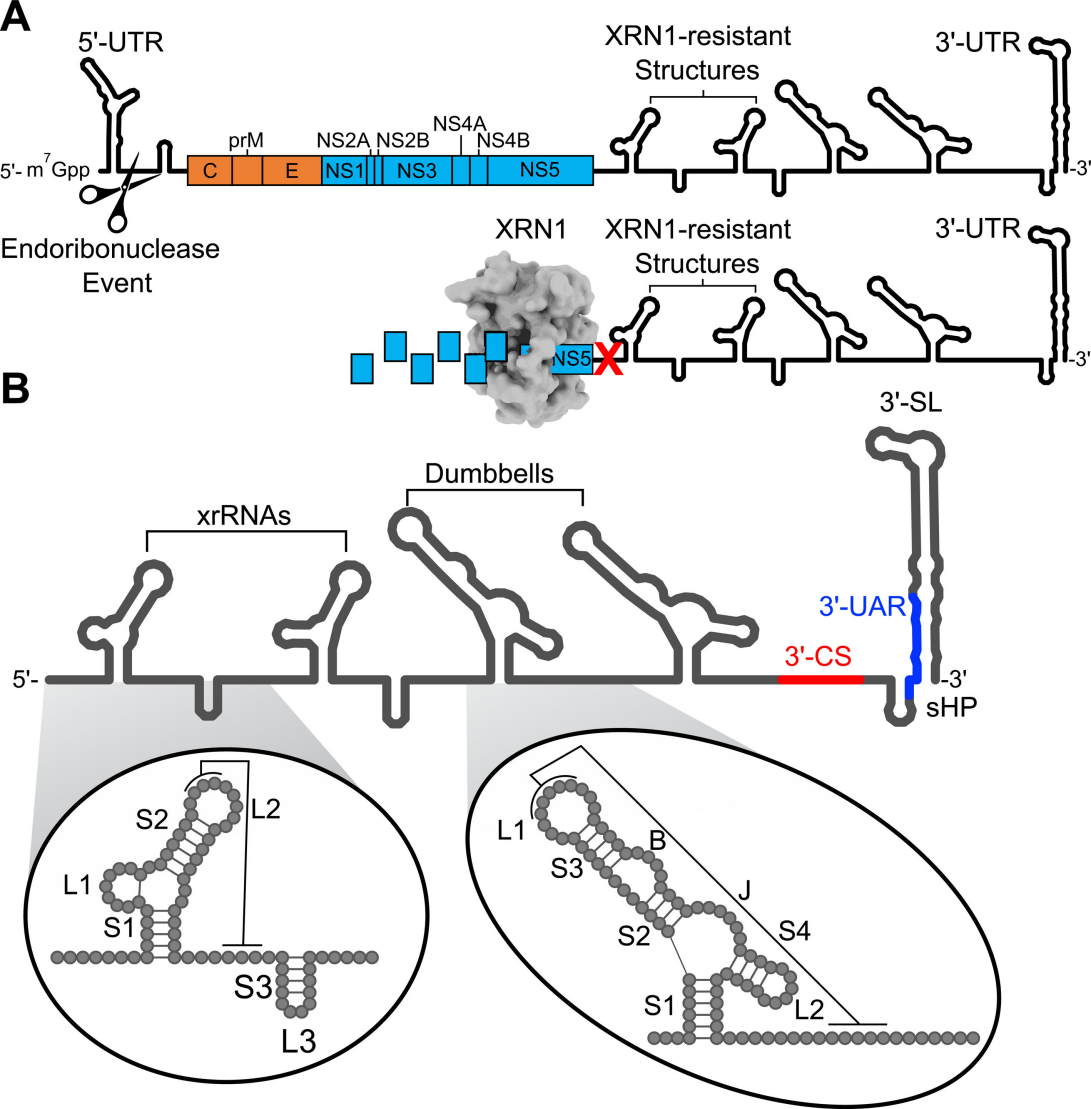
The highly structured flavivirus subgenomic RNAs (sfRNAs) are formed by incomplete digestion by XRN1. (**A**) Schematic of sfRNA biogenesis by the cellular XRN1 enzyme. Following an endonuclease event (depicted by scissors), XRN1 associates with monophosphorylated RNA and degrades gRNA from 5′ to 3′. XRN1 stalls and disassociates at xrRNA RSEs (red X). Cartoon illustrations depicting the 5′ and 3′ UTR flanking coding regions. Orange and blue open reading frames indicate structural and nonstructural genes, respectively. Abbreviations: C, capsid; prM, pre-membrane; E, envelope; NS, nonstructural. (**B**) Structure of flavivirus 3′-UTR/sfRNA RSEs. The 3′ cyclization (CS) and 3′ upstream AUG region (UAR) regions are highlighted in red and blue, respectively. Insets indicate labeling schemes for the xrRNA and DB structures. Abbreviations: S, stem; L, loop; B, bulge; J, junction.

Members of the family *Flaviviridae* possess a ~10 kb positive-sense single-stranded RNA genome that contains a single open reading frame flanked by untranslated regions (UTRs) comprising conserved RNA structural elements (RSEs). During flavivirus replication, ~0.5 kb subgenomic RNAs corresponding to the 3′-UTR are produced by incomplete digestion of the genome by the host 5′−3′ exoribonuclease enzyme XRN1 (8). XRN1 degrades genomic RNA from 5′ to 3′ but stalls and disassociates at one of the XRN-resistant structures (xrRNAs) located early in the 3′-UTR (8–10). This results in the accumulation of small non-coding RNAs comprising 3′-UTR RSEs (Fig. 1A). The accumulation of these small subgenomic flavivirus RNAs (sfRNAs) enhances viral fitness in mammalian cells by inhibiting the interferon response (11, 12), preventing apoptosis (13), and counteracting RNAi (14). Furthermore, viruses unable to produce sfRNAs are highly attenuated in animal models (8, 15). While of long-standing interest, elucidating relationships between sfRNA structure and biological function in the native cellular context has only become more recently tractable with methodological advances in high-throughput RNA probing strategies (16, 17).

Phylogenic analysis of flavivirus 3′-UTRs reveals multiple distinct clades with predicted 3′-UTR RNA structures within each clade characterized by conserved RSEs (18–22). For mosquito-borne flaviviruses (MbFVs), the 3′-UTR is composed of three primary RSEs: the class1a xrRNA (23), dumbbell (DB), and terminal 3’-stem loop (3′-SL) (Fig. 1B). Additionally, the 3′-UTR has been subject to duplication events leading to some viruses that contain multiple xrRNAs and DBs (20, 24). Duplication of these RSEs in some MbFVs appears to facilitate host adaptation and may have differing fitness effects depending on the host (20).

Selective 2′-hydroxyl acylation analyzed by primer extension (SHAPE) chemistries have been used to determine the secondary structures of full-length sfRNAs and isolated 3′-UTR RSEs for the prototypical flavivirus members (25–29). Furthermore, SHAPE reactivities have been reinforced by crystal structures of individual RSEs from various flaviviruses (21, 30–33). However, as these studies were largely performed outside of the context of infection, the 3′-UTR RNA secondary structure in the cellular environment remains understudied. Furthermore, given that sfRNAs and viral genomic RNA (gRNA) are localized in distinct cytoplasmic structures during infection (34), sfRNAs may possess differential structural dynamics than the gRNA 3′-UTR.

In this study, we examined sfRNA secondary structure dynamics for a panel of MbFVs by applying SHAPE-MaP to XRN1-cleaved *in vitro*-transcribed RNA corresponding to the 3′-UTR and to sfRNAs from flavivirus-infected cells. We then used deltaSHAPE and difference SHAPE calculations to compare the data obtained in each context. Our results do not support differences in RNA structure but rather reveal differences in the extent of SHAPE reactivity between *in vitro* and in-cell conditions that are consistent among MbFVs. These observations suggest possible sites for host or viral interactions that are transient in nature.

## RESULTS

### sfRNA accumulation peaks 24–48 h post-infection

**Fig 2:**
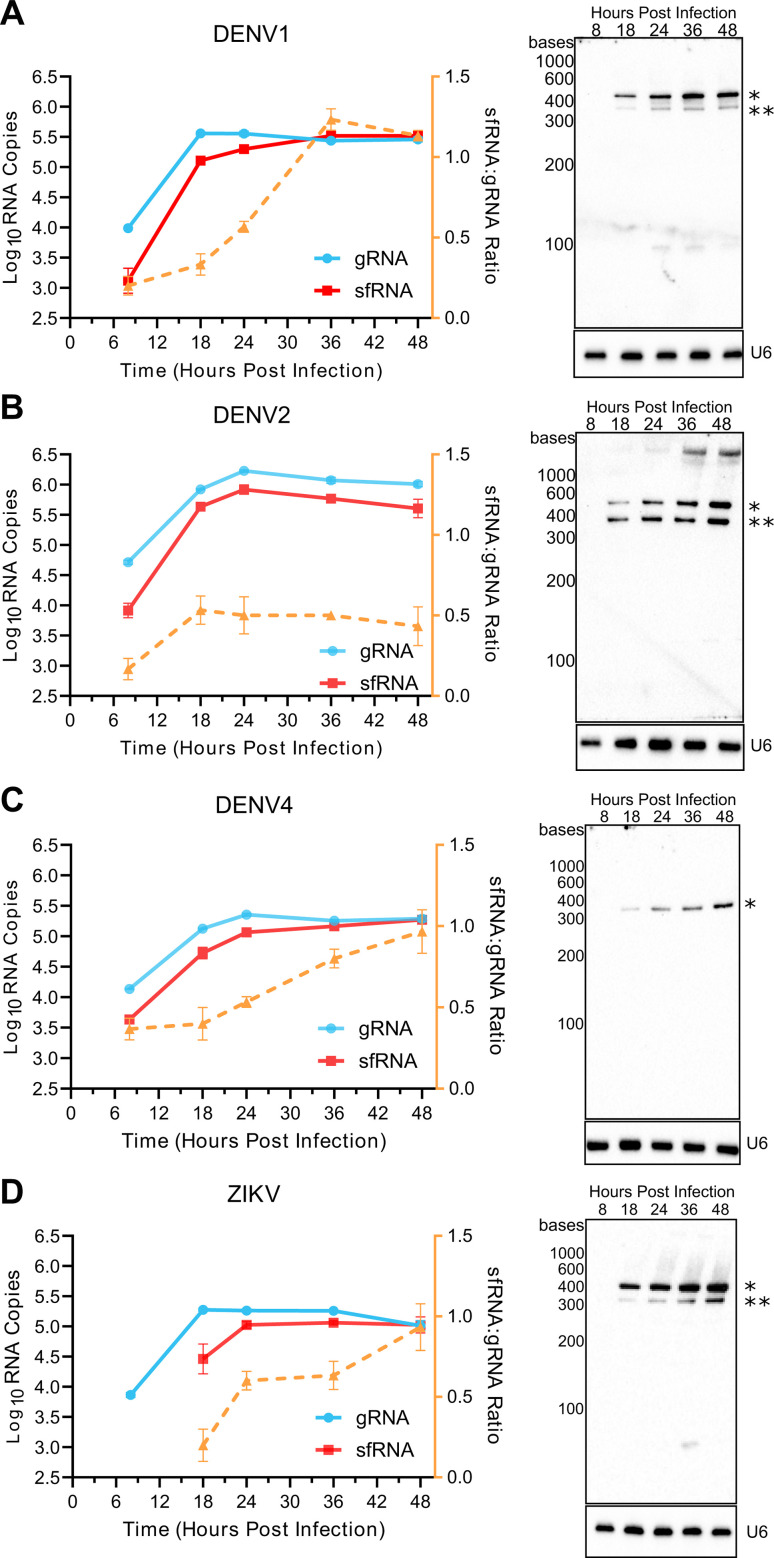
Flavivirus sfRNA accumulation peaks between 24 and 48 hpi. Digital droplet PCR quantification (left) of gRNA (blue), sfRNA (red), and the sfRNA:gRNA ratio (orange) for (**A**) DENV1-, (**B**) DENV2-, (**C**) DENV4-, and (**D**) ZIKV-infected A549 cells. Data shown are mean ± SEM of three biological replicates. Northern blot analysis (right) of total RNA from flavivirus-infected A549 cells. sfRNA1 (*) and sfRNA2 (**) are indicated. The U6 snRNA was used as a loading control.

To define the sfRNA accumulation kinetics of MbFV, we applied digital droplet PCR to quantify gRNA and sfRNA during infection. We examined gRNA and sfRNA copy numbers from total RNA from infected A549 cells at 8, 18, 24, 36, and 48 h post-infection (hpi) from a panel of MbFV, including the prototypical dengue virus serotype 2 (DENV2), dengue virus serotype 1 (DENV1), dengue virus serotype 4 (DENV4), and Zika virus (ZIKV). All viral infections were carried out at a multiplicity of infection (MOI) of 2 plaque-forming units (PFU)/cell. Genome copy number peaked at 18 hpi for DENV1 and ZIKV, while DENV2 and DENV4 lagged behind the other flaviviruses, with gRNA approaching peak accumulation at 24 hpi (Fig. 2). sfRNA accumulated more slowly than gRNA for DENV1, DENV4, and ZIKV, while sfRNA levels reached a plateau at the same time point as gRNA for DENV2. sfRNA copy numbers were lower than gRNA levels for all viruses at the 8, 18, and 24 h time points, while sfRNA to gRNA copy numbers were comparable at later time points for DENV1, DENV4, and ZIKV. Notably, DENV2 sfRNA copy numbers never reached gRNA levels during the time course (Fig. 2). We observed peak sfRNA accumulation for all viruses between 24 and 48 hpi.

We also confirmed sfRNA accumulation using northern blot analysis and observed a similar trend of sfRNA reaching peak accumulation between 36 and 48 hpi. This approach also enabled visualization of distinct sfRNA species, including full-length sfRNAs (sfRNA1) and degradation intermediates (sfRNA2), which correspond to XRN1 stalling at the first or second xrRNA structure in DENV1, DENV2, and ZIKV (Fig. 2A, B and D). Only sfRNA1 was observed for DENV4 due to its single xrRNA (Fig. 2C). From these results, we conclude that accumulation of gRNA and sfRNA are temporally shifted during infection in A549 cells.

### sfRNA secondary structure in cells recapitulates secondary structure *in vitro*

**Fig 3:**
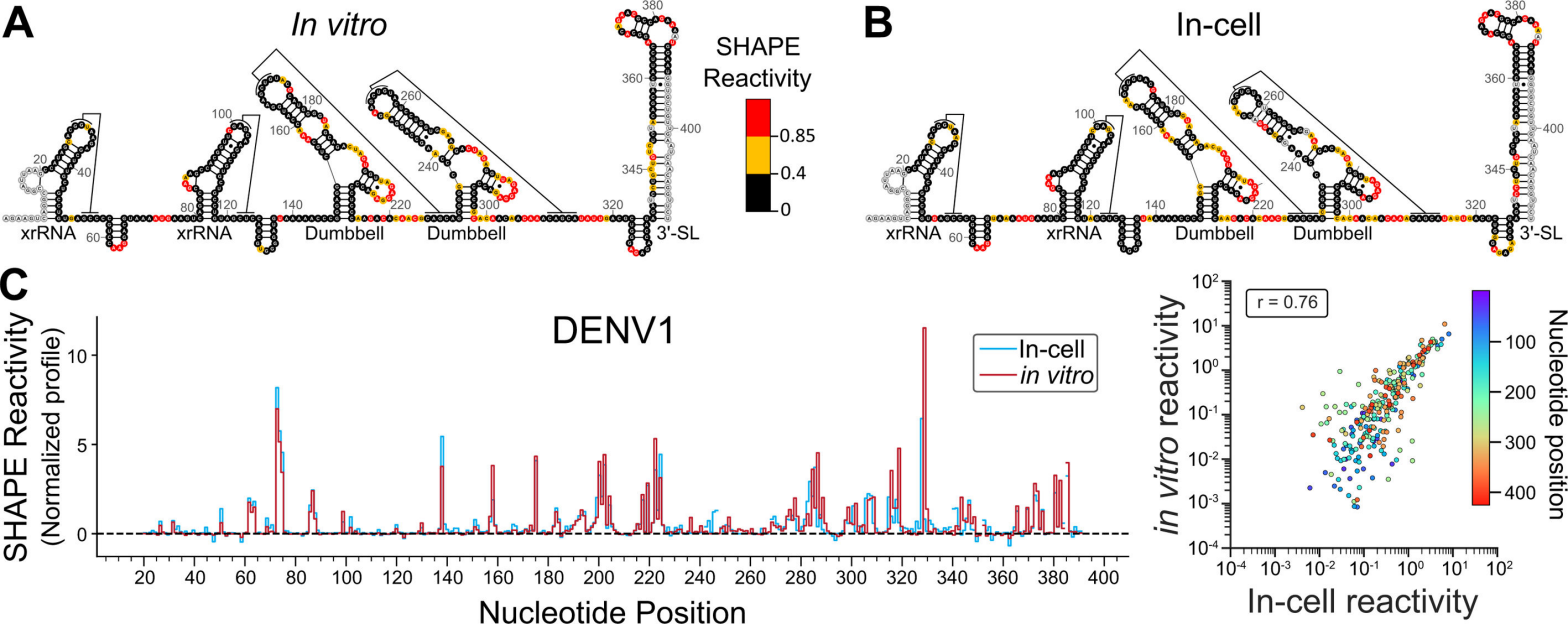
DENV1 sfRNA nucleotide SHAPE reactivity is highly similar *in vitro* and in virus-infected A549 cells. Normalized (**A**) *in vitro* and (**B**) in-cell SHAPE reactivity mapped to the predicted secondary structure of the DENV1 sfRNA. Predicted pseudoknot interactions in the xrRNA and DBs are indicated. Moderate and highly reactive nucleotides are indicated by yellow and red fill, respectively. Undetermined SHAPE reactivity is denoted by gray-outlined nucleotides. (**C**) Left: *in vitro* (red) and in-cell (blue) normalized SHAPE reactivity plotted against nucleotide position. Right: Pearson correlation analysis comparing *in vitro* and in-cell normalized SHAPE reactivity. Individual nucleotides are represented by a single point, and the color is indicative of the nucleotide position in the RNA. The Pearson correlation coefficient (*r*) is also reported.

**Fig 4:**
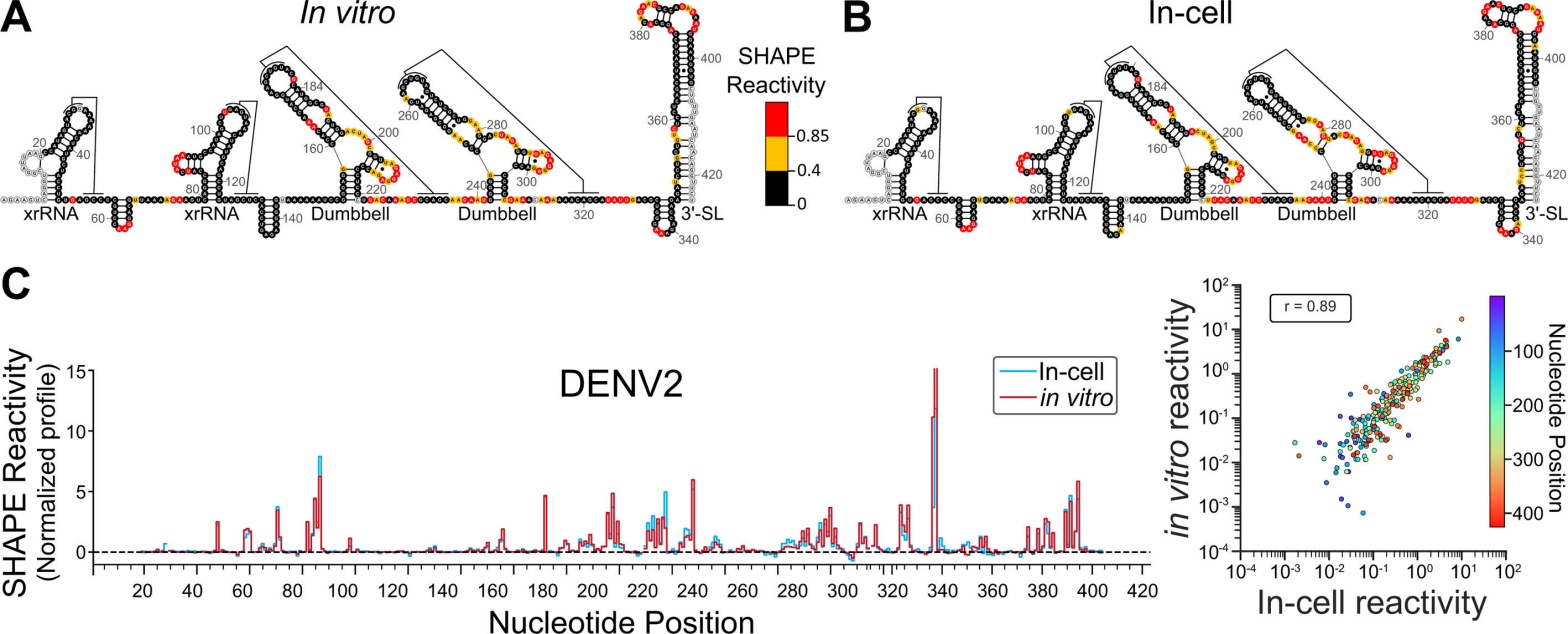
DENV2 sfRNA nucleotide SHAPE reactivity is highly similar *in vitro* and in virus-infected A549 cells. Normalized (**A**) *in vitro* and (**B**) in-cell SHAPE reactivity mapped to the predicted secondary structure of the DENV2 sfRNA. Predicted pseudoknot interactions in the xrRNA and DBs are indicated. Moderate and highly reactive nucleotides are indicated by yellow and red fill, respectively. Undetermined SHAPE reactivity is denoted by gray-outlined nucleotides. (**C**) Left: *in vitro* (red) and in-cell (blue) normalized SHAPE reactivity plotted against nucleotide position. Right: Pearson correlation analysis comparing *in vitro* and in-cell normalized SHAPE reactivity. Individual nucleotides are represented by a single point, and the color is indicative of the nucleotide position in the RNA. The Pearson correlation coefficient (*r*) is also reported.

**Fig 5:**
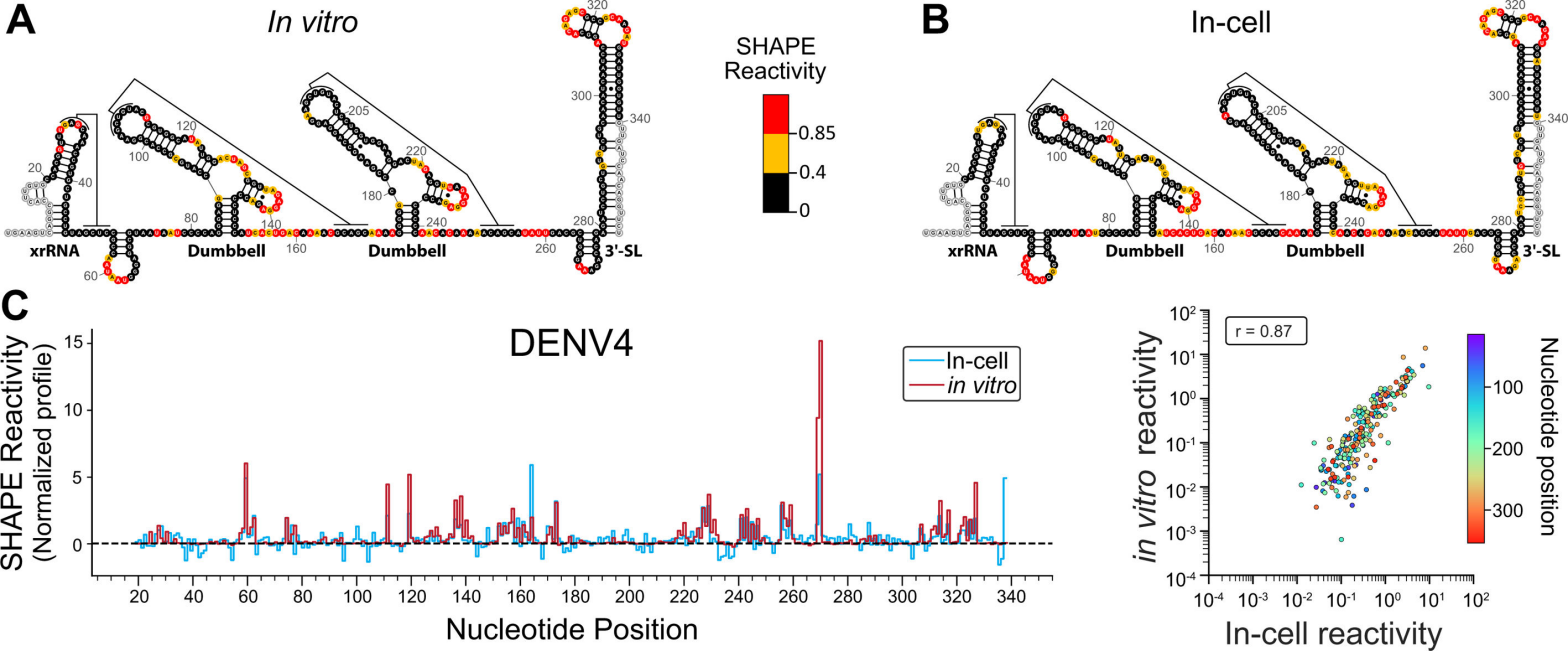
DENV4 sfRNA nucleotide SHAPE reactivity is highly similar *in vitro* and in virus-infected A549 cells. Normalized (**A**) *in vitro* and (**B**) in-cell SHAPE reactivity mapped to the predicted secondary structure of the DENV4 sfRNA. Predicted pseudoknot interactions in the xrRNA and DBs are indicated. Moderate and highly reactive nucleotides are indicated by yellow and red fill, respectively. Undetermined SHAPE reactivity denoted by gray-outlined nucleotides. (**C**) Left: *in vitro* (red) and in-cell (blue) normalized SHAPE reactivity plotted against nucleotide position. Right: Pearson correlation analysis comparing *in vitro* and in-cell normalized SHAPE reactivity. Individual nucleotides are represented by a single point, and the color is indicative of the nucleotide position in the RNA. The Pearson correlation coefficient (*r*) is also reported.

**Fig 6:**
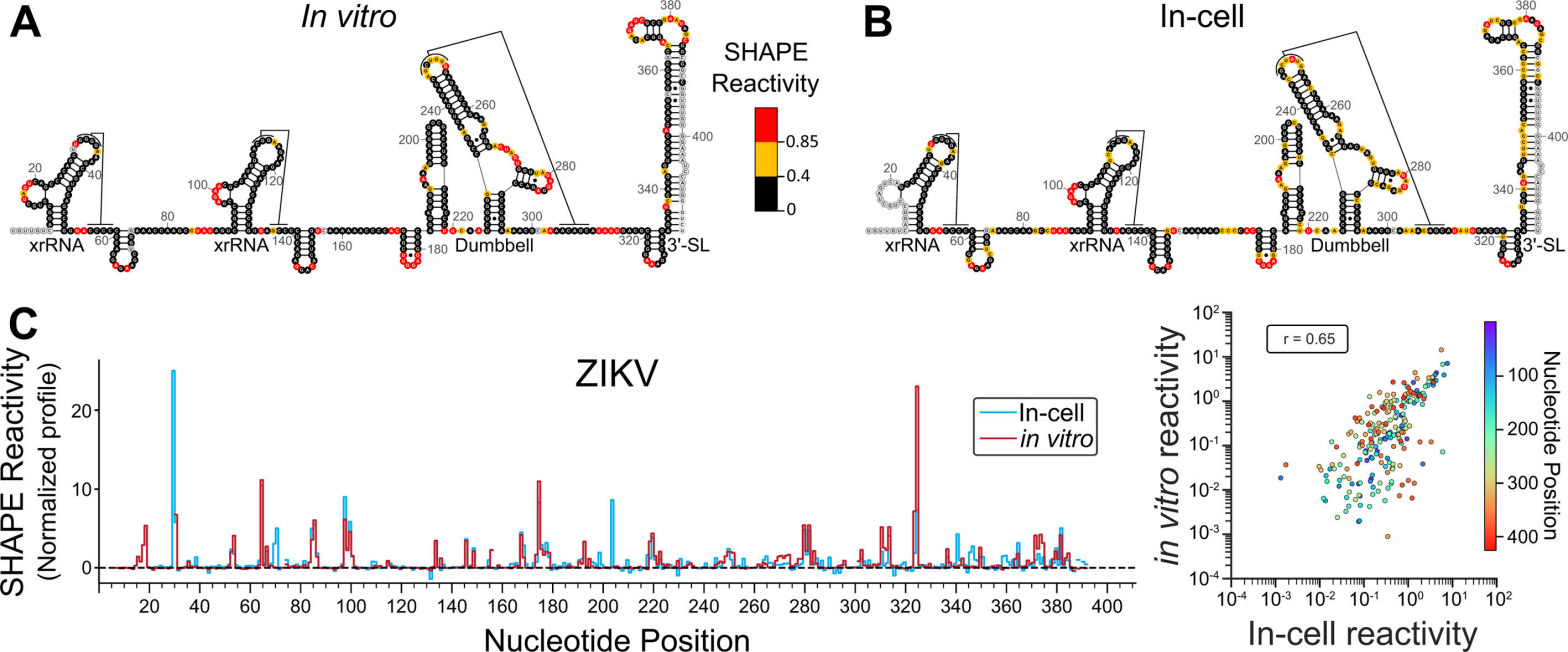
ZIKV sfRNA nucleotide SHAPE reactivity is highly similar *in vitro* and in virus-infected A549 cells. Normalized (**A**) *in vitro* and (**B**) in-cell SHAPE reactivity mapped to the predicted secondary structure of the ZIKV sfRNA. Predicted pseudoknot interactions in the xrRNA and DBs are indicated. Moderate and highly reactive nucleotides are indicated by yellow and red fill, respectively. Undetermined SHAPE reactivity is denoted by gray-outlined nucleotides. (**C**) Left: *in vitro* (red) and in-cell (blue) normalized SHAPE reactivity plotted against nucleotide position. Right: Pearson correlation analysis comparing *in vitro* and in-cell normalized SHAPE reactivity. Individual nucleotides are represented by a single point, and the color is indicative of the nucleotide position in the RNA. The Pearson correlation coefficient ( *r*) is also reported.

To define the sfRNA secondary structural landscapes, we used SHAPE-MaP to compare *in vitro*-transcribed sfRNAs to sfRNAs from A549 cells infected with the same panel of MbFVs. These MbFVs represent variability in 3′-UTR RSE content: DENV1 and DENV2 possess two xrRNAs, two DBs, and the 3′-SL; DENV4 has the same complement of RSEs, with the exception that it only carries one xrRNA; and ZIKV has two xrRNAs, one DB, a structure referred to as a pseudo-dumbbell, and the 3′-SL (19, 22). *In vitro* and in-cell SHAPE-MaP (35) analyses are shown for DENV1, DENV2, DENV4, and ZIKV in Fig. 3 to 6 respectively.

We first performed SHAPE-MaP on *in vitro-*transcribed, XRN1-treated sfRNAs (Fig. S1) and mapped normalized SHAPE reactivity to the predicted sfRNA secondary structures for DENV4, DENV1, DENV2, and ZIKV (Fig 3A through 6A respectively). The results for DENV2 and ZIKV compare well with previously published SHAPE reactivity data (25, 26). While equivalent analyses have not been performed previously for DENV1 and DENV4, the *in vitro* SHAPE reactivity of these sfRNAs also correlates well with that previously reported for DENV2. This outcome was expected based on sequence and structural conservation among MbFV RSEs. However, for DENV4, some novel features were apparent: we observed notable reactivity in the predicted xrRNA pseudoknot loop region (L2), indicating that these interactions may be transient. Additionally, we observed low reactivity at the 3′ end of the DENV4 xrRNA L3 loop and the B region for the 3′-DB, indicating inaccessibility of these nucleotides to SHAPE reactivity due to additional nucleotide interactions or greater stability of these regions.

*In vitro* SHAPE probing does not consider the native cellular environment, in which RNA and/or protein binding partners could alter the SHAPE reactivity observed for isolated sfRNAs. To examine potential differences due to the cellular environment, we therefore compared our *in vitro* results with SHAPE-probed sfRNAs extracted from flavivirus-infected A549 cells. A549 cells were infected with MbFVs at an MOI of 2 PFU/cell. Flavivirus-infected cells were treated with SHAPE reagent (2A3) at the time of peak sfRNA accumulation, as previously determined (Fig. 2): 24 hpi for ZIKV and 48 hpi for DENV1, DENV2, and DENV4. SHAPE-MaP was performed on sfRNAs, and reactivity was mapped to the predicted secondary structure as before (Fig. 3B through 6B respectively). Overall, SHAPE reactivity profiles of sfRNAs from infected cells correlated well with those for *in vitro* transcribed RNAs (Fig. 3C through 6C respectively). We did, however, observe lower than expected correlation (*r* = 0.49) among the in-cell ZIKV replicates (Fig. S2). Additionally, comparisons of mutation rates between *in vitro* and in-cell data sets were relatively consistent (Fig. S3). Skyline plots comparing *in vitro* versus in-cell normalized reactivity reveal moderate per-nucleotide differences. However, despite these subtle differences for individual nucleotides, under both conditions, SHAPE reactivity was consistent with the predicted RSEs. Thus, in-cell SHAPE reactivities correlate well with those determined *in vitro*, revealing that production of sfRNAs within cells does not impose durable changes on the formation of RSEs relative to those seen *in vitro*.

### Comparative analyses reveal changes in the extent of sfRNA SHAPE reactivity

To detect nuanced differences in mapped SHAPE reactivity between *in vitro* and in-cell conditions, we employed two established methods to analyze differences between these contexts: difference SHAPE (36) and deltaSHAPE (37). Difference SHAPE reports increased or decreased in-cell reactivity at the single nucleotide level by subtracting the normalized *in vitro* SHAPE reactivity from the normalized in-cell reactivity. deltaSHAPE, meanwhile, analyzes statistical differences in SHAPE reactivity using a sliding window to identify regions of difference. As expected, these methodologies did not reveal regions of extensive structural difference between *in vitro* and in-cell derived sfRNAs. However, patterns of localized increased or decreased relative SHAPE reactivity were identified that were consistent across viruses.

**Fig 7:**
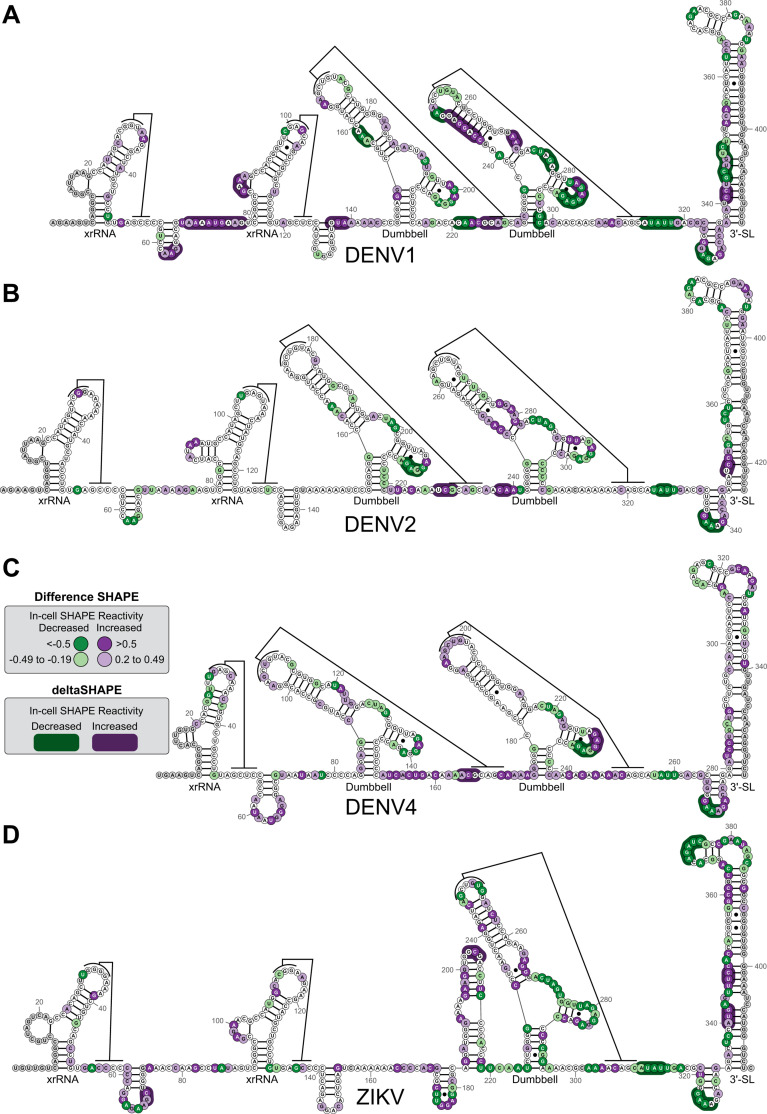
Difference SHAPE and deltaSHAPE analysis of flavivirus sfRNAs reveals consistent, subtle differences between *in vitro* and in-cell SHAPE reactivity. Difference SHAPE and deltaSHAPE mapped to the predicted secondary structures of the (**A**) DENV1, (**B**) DENV2, (**C**) DENV4, and (**D**) ZIKV sfRNAs. Increased or decreased in-cell reactivity is annotated by filled circles in shades of purple (0.20–0.49, or ≥0.50) or green (≤−0.50, or −0.49 to −0.19), respectively. No difference between *in vitro* and in-cell is denoted by white-filled nucleotides. Undetermined SHAPE reactivity is denoted by gray-filled nucleotides. In-cell enhancements and protections reported by deltaSHAPE are denoted by outline shaded regions in dark purple and green, respectively.

Generally, in-cell reactivity was higher than that observed *in vitro* for the flexible nucleotide linkers between RSEs, particularly between the DBs for DENV1, DENV2, and DENV4 (Fig. 7). Additionally, difference SHAPE revealed that the flexible nucleotides in the J region of the DBs had observable decreases in the extent of in-cell SHAPE reactivity. This was seen for all DBs except the DENV1 5′-DB. Consistent among all four viruses was a marked difference in the SHAPE reactivity in the DB S4–L2 region. Specifically, decreased in-cell SHAPE reactivity was seen in this region, and this difference was significant by deltaSHAPE for at least one DB for DENV1, DENV2, and DENV4. We also noted differences in SHAPE reactivity in the lower stem region of the 3′-SL. Finally, we observed significant protection in the small hairpin (sHP) and consistently decreased SHAPE reactivity in the unpaired nucleotides upstream of the sHP.

Together, these results reveal consistent, identifiable differences in the extent of in-cell SHAPE reactivity compared to *in vitro* across the MbFV panel with diverse RSE structural compositions. Importantly, these differences do not suggest large-scale changes in RSE structure. Rather, these differences indicate that the cellular environment gives rise to conditions that alter nucleotide modification by the SHAPE reagent and may thus reflect sites of RNA-RNA, RNA-protein, or other interactions.

## DISCUSSION

We investigated sfRNA production in infected cells and the sfRNA secondary structural dynamics using SHAPE-MaP under *in vitro* and in-cell conditions. We report that sfRNA levels in A549 cells are generally less than gRNA levels, with sfRNA levels reaching a plateau between 24 and 48 hpi. Our *in vitro* SHAPE-MaP results match those previously published for DENV2 and ZIKV and map well to the predicted secondary structure for DENV1 and DENV4. Similarly, SHAPE reactivity from flavivirus-infected A549 cells recapitulated the structural conservation with no major differences in RNA structure relative to *in vitro* conditions. Using two different SHAPE comparison methods, however, differences were observed in the extent of regions of local SHAPE reactivity, some of which were consistent among the viruses tested. These differences highlight the dynamic nature of these RNAs in cells, possibly due to inter-molecular interactions with RNAs or proteins. Our work confirms that the highly stable RSEs in the sfRNAs, including the xrRNAs, DBs, and 3′-SL, remain intact in cells, confirming the previous *in vitro* structural studies. This study provides added depth by showcasing the sfRNA regions that may be subject to nucleic acid or protein interactors in cells. As such, this work provides a foundation for future exploration of the sfRNA function in the cellular environment.

Several reports have used northern blot analysis and quantitative PCR to quantify gRNA and sfRNA in infected cells or mosquitoes (8, 11, 12, 15, 25, 31, 38–40). These reports demonstrate higher levels of sfRNAs compared to gRNAs, with sfRNA:gRNA ratios ranging from 10:1 to >100:1. In contrast, we observed sfRNA:gRNA ratios ≤1.0. This difference may relate to our use of A549 cells, as prior reports probed RNA levels in infected mosquitoes, mosquito cells, or mammalian cell lines such as BHK-21 or HUH-7 cells. A recent study examining sfRNA production and structure in Powassan virus, a tick-borne flavivirus which possesses an evolutionarily distinct class 2 xrRNA structures (23), demonstrated that infected Vero cells produced considerably lower levels of sfRNA compared to gRNA (32). This example highlights diversity in sfRNA kinetics among flaviviruses, although it is not representative of an MbFV. It is likely that the kinetics and efficiency of sfRNA production are dependent on the host system and/or viral species.

Flavivirus DBs are important structural elements of the flavivirus genome. Recent work has demonstrated roles for both dengue virus DBs in viral replication within human and mosquito hosts, with the 3′-DB participating in long-range interactions with the 5′-UTR during genome cyclization (20). Graham and colleagues demonstrated that mutation of the ZIKV DB decreases sfRNA production and sfRNA stability during infection (41). Additionally, Funk and colleagues showed that removal of West Nile virus 5′-DB decreased sfRNA levels in infected cells (15). Furthermore, DENV live attenuated vaccine constructs containing 30 nucleotide deletions (Δ30) in the S3 and L1 regions of the 3′-DB result in significant attenuation and decreased sfRNA production (42, 43). These studies highlight the importance of the DB for the replication of genomic RNA but also in sfRNA biogenesis. Flavivirus DBs, in general, show a remarkable level of structural conservation (33). Interestingly, the areas we found with consistent differences in SHAPE modification between *in vitro* and cellular environments (J and S4–L2; Fig. 4) are known to have high nucleotide conservation among MbFVs (33). Consistent with this evolutionary conservation, the relative nucleotide protection that we observed in cells may indicate that these regions are involved in weak or transient protein:RNA or RNA:RNA interactions during infection. These interactions may implicate binding partners for sfRNA DBs that are important for sfRNA biogenesis, persistence, or function during infection. Both viral and host proteins have been observed to bind viral 3′-UTR RSEs, but the specific binding sites for these proteins are unknown (44). Further testing could include previously identified DB mutants such as the Δ30 vaccine strains. However, considering that these mutants exhibit decreased sfRNA levels, evaluation by in-cell SHAPE will likely require workflows that can achieve high levels of sfRNAs. Our comparative analyses showing consistent reactivity changes, particularly in the 3′-DB, may provide a framework for identifying protein binding sites within the 3′-UTR.

Genome cyclization during replication is accomplished predominantly via two long-range interactions between elements of the 5′-UTR and 3′-UTR: the upstream AUG region (UAR; a 5′-UTR sequence base-pairs with 3′-UTR nucleotides at the 3′ end of sHP and the 5′ portion of the 3′-SL) and the cyclization sequence (CS; the 5′-CS sequence interacts with the complementary 3′-UTR sequence upstream of sHP) (45, 46). Disruption of these regions, including the sHP stem, alters the balance between linear and circular gRNA and reduces replication (47). Our structure probing demonstrated reactive nucleotides throughout the 3′-SL stem structure, particularly in the 3′-UAR (Fig. 3 through 6). This result recapitulated previous reports describing SHAPE probing of other flavivirus 3′-SL, and our results are supported by the fact that this region, particularly the 3′-UAR, is conformationally flexible (48, 49). We also observed relative in-cell protection upstream of the sHP (3′-CS region), in the sHP loop, and in the 3′-UAR regions using both difference SHAPE and deltaSHAPE (Fig. 7). While the sHP loop is not predicted to form long-range interactions during cyclization, the observed enhanced and/or protected nucleotides in the CS and UAR regions do participate in complementary base pairing with the 5′-UTR. These differences in the nucleotide SHAPE reactivity might be due to transient protein interactions taking place during infection or complementary interactions with the 5′-UTR. One possible explanation might be physical interactions between sfRNAs and the 5′-UTR of genomic RNAs, which would require further investigation.

There are some limitations to our study that are important to consider. First, although we enriched for sfRNAs to achieve at least a 10:1 ratio of sfRNA to genomes, full exclusion of gRNA from the in-cell preparations analyzed by SHAPE is not technically feasible. The gRNA 3′-UTR will therefore have made a modest contribution to the levels of SHAPE reactivity reported for in-cell samples. Second, we assume roughly equivalent nucleotide acetylation of the SHAPE reagent in both the *in vitro* and in-cell conditions. Limitations on the uptake of the SHAPE reagent by the cell would result in overall lower nucleotide acetylation in cells compared to *in vitro*. This phenomenon has been observed for some SHAPE reagents (50). To mitigate this concern, we chose the recently described 2A3 reagent because it is highly effective in various mammalian and prokaryotic cells (51).

Interventions that limit the expression or accumulation of sfRNAs during infection would be valuable. Live vaccine candidates that achieve attenuation through mutation of the 3′-UTR, specifically through deletion of portions of the 3′-DB, are currently in various stages of development (52–54). Construction of live flavivirus vaccines with blocked or attenuated sfRNA expression could provide promising candidates for future flavivirus vaccine development (55). Additionally, due to their conservation, the 3′-UTR RSEs are good candidates for therapeutics, and antisense oligonucleotides targeting the 5′- and 3′-UTRs have shown promise to limit flavivirus infection in cells and in animals (56, 57). Flavivirus RSEs could also be targeted by small molecules to disrupt RNA structures (e.g., xrRNA and DB) to limit sfRNA production or reduce replication during infection. To capitalize on these opportunities, a deeper understanding of the flavivirus 3′-UTR RSE structural and interaction dynamics and their roles during infection is needed. Our work confirms that sfRNAs fold as expected in cells during infection and provides a foundation for future studies investigating the function of sfRNAs.

## MATERIALS AND METHODS

### Cells

A549 cells (CCL-185) were obtained from the American Tissue Culture Collection. Cells were verified to be mycoplasma free using the PlasmoTest Detection kit (Invivogen, rep-pt1) and were maintained in DMEM (Gibco 11995073) supplemented with 10% fetal bovine serum (Gemini R&D Systems Catalog number, S11550) and Normocin (Invivogen, ant-nr-2).

### Viruses

Molecular clones of DENV2 (strain 16681) and ZIKV (strain H/PF/2013) have been previously described and were a gracious gift of Ralf Bartenschlager (58, 59). Isolates of WHO reference strains for DENV1 (strain DV1-WP75) and DENV4 (strain DV4-H24) were generously provided by Dr. Matthew Collins (Emory University). For molecular clones, virus stocks were prepared as previously described by electroporation of Vero E6 cells with *in vitro* transcribed viral RNA; harvesting occurred 4–8 days after electroporation (60). For amplification of isolates or stocks from molecular clones, VeroE6 or C6/36 cells were infected at an MOI of 0.01 PFU/cell, and supernatants were harvested 2–8 days after infection, beginning when cells showed cytopathic effect. Extracellular virus titers were determined by plaque assay in Vero E6 cells using a minimum essential medium (Gibco, 41090036) overlay containing 1.0% carboxymethylcellulose.

### Virus infections and RNA extraction

A549 cells were seeded in 12-well plates at a density of 3 × 10^5^ cells/well. Twenty-four hours post-seeding, cell culture medium was removed, and cells were infected with diluted virus (200 µL/well) at an MOI of 2 PFU/cell for 1 h at 37°C with continuous rocking. Supernatant was removed and replaced with complete growth medium (1 mL/well). At 8, 12, 24, 36, and 48 hpi, growth media were removed, and cells were lysed with buffer RLT from the RNeasy Plus RNA isolation kit (Qiagen, 74136) and frozen at −80°C. All lysates were thawed, and RNA was extracted according to the manufacturer’s protocol.

### Northern blot analysis

Total RNA (1 µg) was separated on a 6% denaturing polyacrylamide gel (Invitrogen, EC6865BOX). Gels were stained in RedSafe (iNtRON Biotechnology, 21141) to visualize RiboRuler Low Range ssRNA marker (Thermo Scientific, SM1831). RNA was transferred to Brightstar-Plus Positively Charged Nylon membrane (Invitrogen, AM10102) at 300 mA for 35 min in 0.5× Tris–borate–EDTA running buffer in an Owl Hep1 Electroblotter (Thermo Scientific, HEP-1). RNA was crosslinked in a UV Stratalinker 1800 (Agilent) using the Autocrosslink mode. Membranes were prehybridized in ULTRAhub Oligo Hybridization buffer (Invitrogen, AM8663) at 42°C for 30 min in a Shake “n” Stack Hybridization Incubator (Thermo Scientific, 6243). RNA was then probed with 5’-biotinylated DNA oligonucleotides (see Table S1 for probe sequences) at a final concentration of 5 nM in ULTRAhub Oligo Hybridization buffer overnight at 42°C. Blots were washed in low-stringency wash buffer (2× SSC [Invitrogen, 15557044] and 0.1% SDS [MilliporeSigma, L3771]) three times at room temperature for 5 min each and then once at 42°C for 30 min. Blots were then developed using the Chemiluminescent Nucleic Acid Detection Module (Thermo Scientific, 89880) following the manufacturer’s recommendations and imaged on a ChemiDoc MP imager (Bio-Rad). The imaged blot was aligned with the RedSafe-stained gel image to obtain appropriate sizing of bands. Blots were stripped by incubating the blot in a minimal amount of boiling stripping solution (0.1× SSC and 0.5% SDS) for 15 min at room temperature. The blot was then hybridized, probed (with U6 loading control), and developed as described above.

### Reverse-transcription and sfRNA quantification by digital droplet PCR

Total RNA (1 µg) was reverse-transcribed in 20 µL reactions using Maxima Reverse Transcriptase (Thermo Scientific, EP0743) with the provided buffer, Ribolock RNase inhibitor (Thermo Scientific, EO0381), dNTPs at a final concentration of 0.5 mM (Agilent, 200415-51), and virus-specific reverse-transcription primer (Table S1). cDNAs were diluted 1:100 with water and used as a template for PCR using the QX200 ddPCR EvaGreen Supermix (Bio-Rad, 1864034) and primers anchored in the NS5 or 3′-UTR regions (Table S1). Droplets were generated using a QX200 Droplet Generator (Bio-Rad, 1864002) and subsequently amplified in a thermocycler using the following conditions: 95°C for 5 min; 40 cycles of 95°C for 30 s, 58°C for 1 min; 4°C for 5 min; 90°C for 5 min; and hold at 4°C. Positive droplets were then detected on the QX200 Droplet Reader (Bio-Rad, 1864003). gRNA copies were determined by the positive droplets from the NS5 primer set. sfRNA copies were calculated by subtracting the NS5 copies from the 3′-UTR copies.

### RNA synthesis

Plasmids encoding the sfRNAs from DENV1 (Western Pacific Strain [AY145121], nucleotides 10,310–10,736), DENV2 (Strain 16681 [NC_001474.2], nucleotides 10,282–10,723), DENV4 (TVP/360 strain [KU513442.1], nucleotides 10,269–10,649), and ZIKV (H/PF/2013 strain [KJ776791.2], nucleotides 10,373–10,807) were purchased from Genscript. sfRNA encoding sequences were subcloned into an *in vitro* transcription plasmid encoding a 3′ Hepatitis Delta Virus ribozyme to ensure production of RNAs with homogeneous 3′ ends (61). Plasmids were purified as previously described, with the exception that ribosomal RNA was removed using Capto Core 700 multimodal chromatography resin (Cytiva, 17548102) (62). The plasmid was linearized using the EcoRV restriction enzyme (New England Biolabs, R3195T) and verified by agarose gel electrophoresis. Typical *in vitro* transcriptions were performed using 200 mM HEPES-KOH (pH 7.5), 28 mM MgCl_2_, 2 mM spermidine, 40 mM DTT, 10 mM rNTPs, 100 µg/mL of linearized template DNA, and 2.5 µL of T7 RNA polymerase (prepared in-house). Reactions were incubated at 37°C for 3 h (63). The pyrophosphate-magnesium precipitate was cleared using 0.2× volume of 250 mM EDTA (pH 8.0). RNA was dialyzed overnight at 4°C in 1× TE buffer (10 mM Tris-HCl [pH 8.0] and 1 mM EDTA) and ethanol precipitated. RNAs were separated using 6% native polyacrylamide gels (Invitrogen, EC6261BOX). RNA bands were visualized using UV shadowing at 254 nm, excised, and eluted using the crush-soak method in 0.3 M sodium acetate (Invitrogen, AM9740), followed by ethanol precipitation. RNA pellets were resuspended in 1× TE, and the concentrations were determined by spectrophotometry.

### 2A3 synthesis

In-house synthesis of 2A3 was performed essentially as previously described (51). Briefly, 552.5 mg of 2-aminopyridine-3-carboxylic acid (MilliporeSigma, A68300) and 648.6 mg of 1,1′-carbonyldiimidazole (MilliporeSigma, 21860) were added to a 5 mL beaker. Anhydrous dimethyl sulfoxide (DMSO) (4 mL, MilliporeSigma, 276855) was added and stirred at high speed continuously for 1 h at room temperature. The solution was then spun at 16,100 × *g* for 2 min. The supernatant was transferred to cryogenic vials and stored at −80°C until use.

### XRN1 treatment of *in vitro*-transcribed sfRNAs

Flavivirus RNA (2 µg) was heated to 95°C for 2 min, then snap cooled on ice. Concentrated folding buffer (final 50 mM Tris-HCl pH 7.9, 100 mM NaCl, 10 mM MgCl_2_, and 1 mM DTT) was then added, and the RNA was heated to 55°C for 10 min, slowly cooled to 25°C at 1°C/min, and then chilled on ice. XRN1 (New England Biolabs, M0338, 2 units) and RppH (New England Biolabs, M0356, 10 units) were then added and incubated at 37°C for 1 h. XRN1 cleavage was verified by electrophoresis on a 6% TBE-urea polyacrylamide gel (Invitrogen, EC6865BOX) stained with RedSafe.

### 2A3 treatment of *in vitro*-transcribed RNA

XRN1-digested RNA (20 µL total) was incubated at 37°C for 5 min. RNA (10 µL) was then DMSO- or 2A3-treated (1 µL) and incubated at 37°C for 15 min. The reaction was quenched by 10 µL of 1 M DTT. RNAs were then purified using Illustra MicroSpin G-25 Columns (Cytiva, 27-5325-01) following the manufacturer’s protocol.

### In-cell 2A3 treatment

A549 cells were flavivirus-infected at an MOI of 2 PFU/cell. 2A3 treatment was performed at 24 hpi (ZIKV) or 48 hpi (DENV1, DENV2, and DENV4). At these time points, cells were healthy enough to undergo SHAPE treatment, and sfRNA levels had plateaued. Cell culture medium was removed, and cells were washed once with 1× PBS. Prewarmed complete cell culture medium (900 µL) mixed with DMSO or 2A3 (100 µL) was used to treat cells for 15 min at 37°C, then neutralized with 1 mL of 1 M DTT. Total RNA was then extracted using TRIzol reagent (Invitrogen, 15596026).

### Enrichment of flavivirus sfRNA

Ribosomal RNA was removed from 5 µg of TRIzol-extracted total RNA using the RiboMinus Eukaryotic System v2 (Invitrogen, A15026) following the manufacturer’s protocol. RNA was then size separated using RNAClean XP beads (Beckman Coulter, A63987). Briefly, concentrated denaturing buffer (final 10 mM Tris-HCl pH 8.0, 2 M urea) was combined with ribosomal RNA-depleted RNA to a total volume of 50 µL then heated to 90°C for 5 min and snap cooled on ice. RNA was then separated with 25 µL (0.5×) RNAClean XP beads. The eluted RNA (Elution 1) was saved, and the supernatant (RNA not bound to beads) was extracted again using 0.5× RNAClean XP beads. The eluted RNA (Elution 2) was saved, and the supernatant was extracted using 1.8× RNAClean XP beads. The eluted RNA (Elution 3) was saved. Each elution was evaluated for sfRNA enrichment using the digital droplet PCR assay previously discussed. Samples were processed for SHAPE-MaP if the sfRNA:gRNA enrichment was >10×.

### SHAPE-MaP library preparation

SHAPE-MaP was performed essentially as previously described (35). DMSO- or 2A3-treated RNA was treated with 4 units of TURBO DNase (Invitrogen, AM2238) with the included buffer for 1 h at 37°C. DNase-treated RNA was then purified using the Monarch Spin RNA Cleanup kit (New England Biolabs, T2030L). RNA was eluted in 13 µL of nuclease-free water. RNA (10 µL) was then combined with 1 µL of 2 µM reverse-transcription primer (Table S1), heated to 65°C for 5 min, then immediately placed on ice. MaP buffer (35) and SuperScript II Reverse Transcriptase (Invitrogen, 18064014) were then added, mixed, and incubated at 37°C for 3 h. The reverse transcriptase was then heat inactivated at 70°C for 5 min, and the cDNA products were purified using Illustra MicroSpin G-25 Columns. Library preparation was performed by first PCR amplifying the sfRNA using Phusion High-fidelity DNA Polymerase (New England Biolabs, M0530L) and flavivirus-specific primers (Table S1) and 5 µL of cDNA template in a 50 µL volume. PCR amplicons (5 µL) were electrophoresed on a 1% agarose TAE gel, and products were visualized using RedSafe nucleic acid staining solution. Libraries were constructed with the Nextera XT DNA Library Preparation Kit (Illumina, FC-131-1096) and Illumina DNA/RNA UD Indexes with 1 ng cDNA (tagmented) as input. The final amplified libraries were purified using AMPure XP bead cleanup (Beckman Coulter, A63882). Libraries were validated by capillary electrophoresis on a TapeStation 4200 (Agilent), pooled at equimolar concentrations, and sequenced with PE100 reads on a NovaSeq 6000 (Illumina), targeting a depth of 500,000 reads per sample.

### Data analysis

Raw sequencing FASTQ files were inputted into the ShapeMapper2 (35, 64) for read alignment, mutation counting, and calculation of normalized nucleotide reactivity using default parameters with a minimum read depth of 5,000. All ShapeMapper2 runs passed the built-in quality control checks. The final data set for each virus included two independent *in vitro* and in-cell replicates. Replicate normalized reactivity was averaged and used in the downstream analysis. Linear regression of replicate data sets was performed using the RNAvigate pipeline (65) with Pearson correlation coefficients of 0.76, 0.89, 0.87, and 0.65 for DENV1, DENV2, DENV4, and ZIKV, respectively. Further correlation analysis performed on ZIKV demonstrated one in-cell replicate (ZIKV_Cell_R1) correlated poorly with the *in vitro* replicates and the other in-cell replicate. We included both in-cell replicates in our final analysis as we could not exclude either in-cell replicate with cause. Comparison of per-nucleotide data was visualized using Skyline plots generated using RNAvigate. Normalized nucleotide reactivity was mapped to the predicted sfRNA secondary structures using RNAcanvas (66). Comparison of *in vitro* and in-cell SHAPE-MaP reactivity was performed using Difference SHAPE (36) and deltaSHAPE (37). The per-nucleotide averaged *in vitro* normalized SHAPE reactivities were subtracted from the in-cell data set. The difference in in-cell reactivity for each nucleotide was then classified as decreased (≤−0.50, or −0.49 to −0.19), no change (−0.20 to 0.19), or increased (0.20–0.49, or ≥0.50). For deltaSHAPE, SHAPE-MaP-generated profiles.txt files were averaged between replicates then used as raw data for the execution of the deltaSHAPE pipeline integrated into the RNAvigate suite using default parameters. Enhanced and protected nucleotides were then mapped to the secondary structure of each sfRNA using RNAvigate and transferred to RNAcanvas. ShapeMapper2 mutation rates were extracted from the profiles file provided by SHAPE-MaP analysis and plotted using GraphPad Prism version 11.0.0 for Windows (https://www.graphpad.com/).

## Data Availability

Sequences are available through the NCBI Sequence Read Archive (https:// www.ncbi.nlm.nih.gov/sra) under BioProject accession number PRJNA1443375.

## SUPPLEMENTAL MATERIAL

Supplemental material jvi.00898-26-s0001.docx Fig. S1 to S3; Table S1.

ASM does not own the copyrights to Supplemental Material that may be linked to, or accessed through, an article. The authors have granted ASM a non-exclusive, world-wide license to publish the Supplemental Material files. Please contact the corresponding author directly for reuse.
