# Supplementary material for: Comparative analysis of flavivirus sfRNA dynamics and secondary structure
Source: J Virol. 2026 Jul 10;100(8):e00898-26. doi: 10.1128/jvi.00898-26 (PMC13483245; doi:10.1128/jvi.00898-26)
Supplement: Supplementary file 1 [file jvi.00898-26-s0001.docx]

**Supplementary Materials**

**Comparative analysis of flavivirus sfRNA dynamics and secondary structure**

Camden R. Bair^a#^, David VanInsberghe^a^, Anice C. Lowen^a^, Graeme L. Conn^b^, Christopher J. Neufeldt^a#^

*^a^Department of Microbiology and Immunology, Emory University School of Medicine, Atlanta, GA, 30322*

*^b^Department of Biochemistry, Emory University School of Medicine, Atlanta, GA, 30322*

#Address correspondence to: [camdenbair@gmail.com](mailto:camdenbair@gmail.com) or [c.neufeldt@emory.edu](mailto:c.neufeldt@emory.edu)

**This file contains:**

Supplementary Fig. S1-S3

Supplementary Table S1

**Supplementary Figures**

**Fig. S1: *in vitro*-transcribed and folded sfRNAs are resistant to XRN1.** sfRNAs used in *in vitro* SHAPE probing experiments were untreated or treated with XRN1 and RppH. RNAs (100 ng) were resolved on a 6% TBE-urea gels and stained with RedSafe.

**Fig. S2: ZIKV in-cell replicates exhibit low correlation.** Pearson correlation analysis of normalized SHAPE reactivity among ZIKV *in vitro* and in-cell replicates. Individual nucleotides are represented by a single point, and the color is indicative of the nucleotide position in the RNA. The Pearson correlation coefficient (r) is also reported.

**Fig. S3: *In vitro and* in-cell sfRNA nucleotide SHAPE mutation rates are similar.** sfRNA mutation rates for *in vitro* and in-cell conditions were calculated with ShapeMapper 2. Boxes represent the 25^th^ to 75^th^ percentiles with the middle line indicating the median. The whiskers extend to the 10^th^ and 90^th^ percentiles. Unmodified (DMSO-treated) or modified (2A3-treated) are indicated by light orange and dark orange boxes, respectively.

**Supplementary Tables**

**Table S1. Sequences of primers used in this study.**

| Primer Name | Sequence 5’ to 3’ | Purpose |
| --- | --- | --- |
| DENV1 Reverse Transcription Primer | AGA ACC TGT TGA TTC AAC AGC AC | Reverse transcription primers for SHAPE-MaP. |
| DENV2 Reverse Transcription Primer | AGA ACC TGT TGA TTC AAC |  |
| DENV4 Reverse Transcription Primer | AGA ACC TGT TGG ATC AAC |  |
| ZIKV Reverse Transcription Primer | GAG ACC CAT GGA TTT CCC CAC |  |
| DENV1 SHAPE AMP Fw | AGA AGT CAG GCT GGA TTA AG | Amplification primers for SHAPE-MaP. |
| DENV1 SHAPE AMP Rv | AGA ACC TGC TGA TTC AAC |  |
| DENV2 SHAPE AMP Fw | AGA AGT CAG GTC GGA TTA AG |  |
| DENV2 SHAPE AMP Rv | AGA ACC TCT TGA TTC AAC AG |  |
| DENV4 SHAPE AMP Fw | TGA AGT CAG TCC ACT TGT G |  |
| DENV4 SHAPE AMP Rv | AGA ACG TGT TGG ATC AAC |  |
| ZIKV SHAPE AMP Fw | TGT TGT CAG GTC TGC TAG TCA GC |  |
| ZIKV SHAPE AMP Rv | AGA CTC ATG GAT TTC CCC ACA CC |  |
| DENV1 NB Probe | /5Biosg/ CTG TGC CTG GAA TGA TGC TGT AGA GAC AGC AGG ATC TCT GG | 5’-Biotinylated oligonucleotide sequences for northern blot probing |
| DENV2 NB Probe | /5Biosg/ CTG TTG ATT CAA CAG CAC CAT TCC ATT TTC TGG CGT TCT GTG CC |  |
| DENV4 NB Probe | /5Biosg/ ACA GCA GGA TCT CTG GTC TTT CCC AGC GTC AAT AT |  |
| ZIKV NB Probe | /5Biosg/ CTC ATG GAG TCT CTG GTC TTT CCC AGC GTC AAT AT |  |
| DENV1 ddPCR gRNA Fw | TCA GCC GTT CCA GTT GAT TGG | Digital droplet PCR primers. |
| DENV1 ddPCR gRNA Rv | ATT GAT GGT GGG CAT GGA TCG |  |
| DENV1 ddPCR UTR Fw | GAA AGC CAC GGT TCG AGC AAG |  |
| DENV1 ddPCR UTR Rv | GGG TAC AGC TTC CCC TGG TG |  |
| DENV2 ddPCR gRNA Fw | ACG GAA GAC ATG CTG ACA GTC TG |  |
| DENV2 ddPCR gRNA Rv | AGC CGC ACC ATT GGT CTT CTC |  |
| DENV2 ddPCR UTR Fw | CCA AGG CGA GAT GAA GCT G |  |
| DENV2 ddPCR UTR Rv | GTC TTT CCC AGC GTC AAT ATG C |  |
| DENV4 ddPCR gRNA Fw | GCT TAC GCC CAG ATG TGG TC |  |
| DENV4 ddPCR gRNA Rv | TGG AAC TGC TGA GCA TAT GGC |  |
| DENV4 ddPCR UTR Fw | GCG TGG CAT ATT GGA CTA GC |  |
| DENV4 ddPCR UTR Rv | ACA GCT TCC TCC TGG CTT CG |  |
| ZIKV ddPCR gRNA Fw | GGA TCT CTC ATA GGG CAC AGA CC |  |
| ZIKV ddPCR gRNA Rv | GCA CTC CAG GTG TAG ACC CTT C |  |
| ZIKV ddPCR UTR Fw | AAG AAG CCA TGC TGC CTG TG |  |
| ZIKV ddPCR UTR Rv | AGC TGA TCT CCA GTT CAG G |  |
